# Dynamic features of water molecules in superconcentrated aqueous electrolytes

**DOI:** 10.1038/s41598-018-27706-5

**Published:** 2018-06-19

**Authors:** Sungho Han

**Affiliations:** CAE Group, Platform Technology Lab, Samsung Advanced Institute of Technology, Suwon, Gyeonggi 16678 Korea

## Abstract

An existence of ions dissolved in water has significant effects on bulk properties of water. Superconcentrated conditions have been recently proposed to provide a new concept of lithium ion batteries in order to overcome limitations for practical applications. In those conditions, water would undergo significant changes in structure and dynamics compared to its bulk properties. However, little is known about water in superconcentrated aqueous electrolytes. Here we study the properties of water in aqueous electrolytes with various salt concentrations via molecular dynamics simulations. We find that new dynamic features of water arise in the limit of an extremely high salt concentration. In particular, we observe a decoupled temporal character of water molecules exhibiting a subdiffusive translation and a diffusive rotation in the superconcentrated condition. Furthermore, we find that the rotational dynamics for each principal axis of a water molecule differently responds to the salt concentration, resulting in an occurrence of anisotropy in the rotation as the salt concentration increases. The superconcentrated environments also invoke new features in the hydrogen-bonding characteristics of water such as an emergence of two time scales in the hydrogen bond dynamics of water with respect to the salt concentration.

## Introduction

Water is one of the most important and ubiquitous materials in nature^1–3^. It exhibits many distinct thermodynamic and dynamical properties compared to other liquids. The unique properties of water generally stem from the forming and breaking of its hydrogen bond (HB) network^4–6^. The number of HBs per water molecule is on average 3.4~3.6 at room temperature for bulk water^6^. The resultant tetrahedral structure of water generates many unusual properties, such as the liquid-liquid phase transition of supercooled water^3,7^. Thus, the hydrogen-bonding ability of water has been considered a key element to explain the uniqueness of water from other liquids and as a result many researches have focused on the characteristic features of the HBs of water^4–6^. When the hydrogen-bonding network of water is disrupted, one finds that water would exhibit special features different from bulk water. Water confined in nanopore geometries is the celebrated example. Restricted spatial freedom by nanoconfinement induces the distorted hydrogen-bonding network, so that nanoconfined water exhibits many different properties from bulk water^6,8,9^. Spontaneous freezing of water at room temperature, distinct from bulk water, has been found in nanoconfined water^10,11^.

In addition to the geometry effects, an existence of ions has salient effects on bulk properties of water. Generally, the properties of solutions of ions in water are of relevance in a wide range of systems, so that understanding the structural and dynamic properties of the solution systems has been of great interest. One of the main issues in this field is how different ions would give perturbations on the HB network of bulk water, thus generating peculiar structural and dynamic properties of water in electrolyte solutions^12–14^. After introducing a concept of “structure making/breaking” induced by ions^15^, the effects of ions on the properties of water have been extensively studied. Some results have been in good agreement with the interpretation, but some have not^12,13^. The salient effects of ions on the dynamics of water are also recognized. Recent studies have shown that ions can enhance or suppress the dynamics of water in the presence of different ions with the subtle change in the structure of water^16,17^. Despite long efforts to reveal the effects of ions on the structure and dynamics of water, many questions still remain open and some of them are subjects of ongoing debates^12,13^.

The applications of solutions of ions in water could extend to lithium ion batteries. For lithium ion batteries, there have been several important issues concerning safe, environmentally friendly and low cost batteries, which are mostly raised by nonaqueous electrolytes. Thus, finding suitable electrolytes for lithium ion batteries has been a big challenge for a long time^18–21^. Water has been one of strong candidates for electrolytes satisfied with most requirements. However, the narrow electrochemical stability of water, namely, the decomposition of a water molecule at 1.23 volts prohibits it from being utilized in lithium ion batteries due to the strong demand of battery operations at higher voltages. Importantly, recent experiments have shown, by presenting the stable operation of lithium ion batteries up to 3 volts, that an extremely high concentration of a lithium bis(trifluoromethane sulfonyl)imide (LiTFSI) salt (called “water-in-salt”) could help to hurdle the obstacle^22–25^.

**Figure 1:**
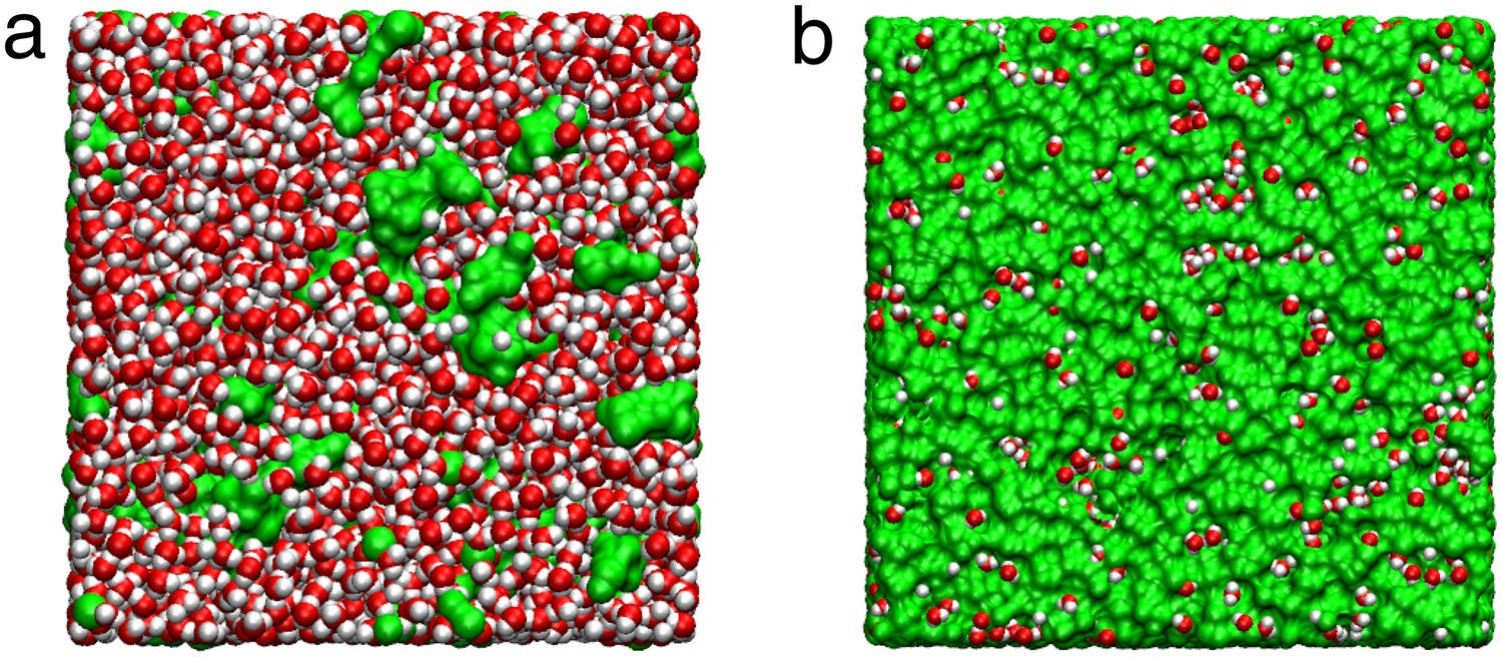
Configurations of electrolytes with low and high salt concentrations. Shown are snapshots of aqueous electrolytes at the salt concentrations of (**a**) 1 M and (**b**) 20 M, respectively. Green domains represent the volumes occupied by cations and anions. Red and white spheres represent oxygen and hydrogen atoms of water molecules, respectively.

The environments surrounding water molecules are quite different from bulk water for the low and high salt concentrations, as shown in Fig. 1. Since the properties of water are critical for understanding aqueous electrolytes and the dynamics of water is significantly influenced by an existence of ions^14,16,17,26^, it is necessary to explore the properties of water in specific conditions of electrolytes. However, systematic studies of water in superconcentrated aqueous electrolytes, to the best of our knowledge, have not been performed so far. Therefore, motivated by the recent experiments^22–24^, we study the dynamics of water in superconcentrated aqueous electrolytes for the first time.

## Results and Discussion

### Translational and rotational dynamics of water molecules

**Figure 2:**
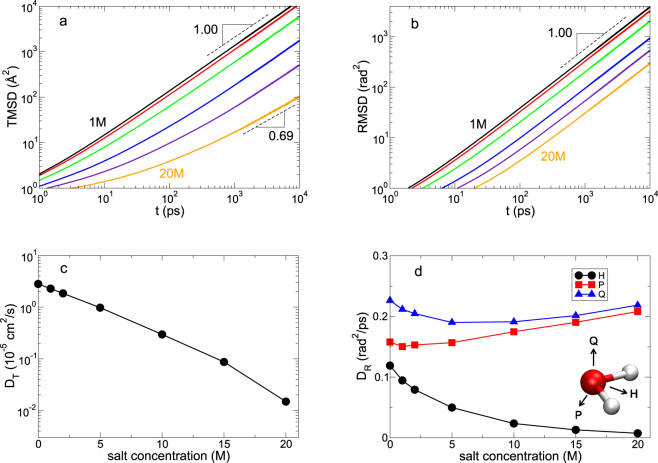
The translational and rotational dynamics of water molecules. (**a**) The translational mean square displacement (TMSD), (**b**) the rotational mean square displacement (RMSD) of the polarization vector $\overrightarrow{H}$, (**c**) the translational diffusion constant *D*_*T*_ and (**d**) the rotational diffusion constant *D*_*R*_. In (**a**) and **(b**), we present the results of the salt concentrations of 1, 2, 5, 10, 15 and 20 M, respectively. In (**c**), *D*_*T*_ at 20 M is not the real translational diffusion constant due to the subdiffusive motion, which is simply the value (divided by 6) corresponding to the slope of TMSD. In (**d**), we present rotational motions of three principal axes represented by $\overrightarrow{H}$, $\overrightarrow{P}$ and $\overrightarrow{Q}$. In (**c**) and (**d**), 0 M denotes bulk.

First, we investigate the dynamics of water by calculating the translational and rotational mean square displacements (TMSD and RMSD). The TMSD is defined as^8,11,27^

1 $$\langle {\rm{\Delta }}{\overrightarrow{r}}^{2}(t)\rangle \equiv \langle \frac{1}{N}\sum _{i=1}^{N}{[{\overrightarrow{r}}_{i}(t+{t}_{0})-{\overrightarrow{r}}_{i}({t}_{0})]}^{2}\rangle ,$$

where $\langle \cdots \rangle$ represents an ensemble average. To obtain the analogue of the RMSD^28–30^, we quantify the rotational motion of a water molecule using the normalized polarization vector $\overrightarrow{H}(t)$ defined as the unit vector connecting an oxygen atom with the midpoint of the line joining two hydrogen atoms (Fig. 2). For a time interval *δt*, the vector $\overrightarrow{H}$ spans the angle $\delta \phi \equiv {\cos }^{-1}[\overrightarrow{H}(t+\delta t)\cdot \overrightarrow{H}(t)]$. We define an angle vector $\delta \overrightarrow{\varphi }$ to be that the magnitude is $|\delta \overrightarrow{\varphi }(t)|=\delta \phi$ and the direction is given by $\overrightarrow{H}(t)\times \overrightarrow{H}(t+\delta t)$. At last, we obtain the desirable angle vector $\overrightarrow{\varphi }(t)$ by summing $\delta \overrightarrow{\varphi }(t)$ over time *t*,

2 $$\overrightarrow{\varphi }(t)={\int }_{0}^{t}dt^{\prime} \delta \overrightarrow{\omega }(t^{\prime} ),$$

where $\delta \overrightarrow{\omega }(t)\equiv \delta \overrightarrow{\varphi }(t)/\delta t$ ^28–30^. It allows us to define a trajectory of the angle vector $\overrightarrow{\varphi }(t)$ to examine the rotational dynamics of a water molecule. Now we are able to define the RMSD similar to the TMSD,

3 $$\langle {\rm{\Delta }}{\overrightarrow{\varphi }}^{2}(t)\rangle \equiv \langle \frac{1}{N}\sum _{i=1}^{N}{[{\overrightarrow{\varphi }}_{i}(t+{t}_{0})-{\overrightarrow{\varphi }}_{i}({t}_{0})]}^{2}\rangle \mathrm{.}$$

For bulk water, the TMSD is characterized by three different time regimes in the relation of ${\mathrm{lim}}_{t\to \infty }\langle {\rm{\Delta }}{\overrightarrow{r}}^{2}(t)\rangle \sim {t}^{\alpha }$: the initial ballistic regime (*α* = 2) followed by a cage regime (*α* = 0) and the diffusive regime (*α* = 1) in the long time. The RMSD is also characterized with the same time evolution in the relation of ${\mathrm{lim}}_{t\to \infty }\langle {\rm{\Delta }}{\overrightarrow{\varphi }}^{2}(t)\rangle \sim {t}^{\beta }$: the initial ballistic regime (*β* = 2), the intermediate cage regime (*β* = 0) and the diffusive regime (*β* = 1) in the long time^29^. A significant dynamic coupling between the translational and rotational motions has been found in bulk water^29,30^.

In Fig. 2, we present the TMSD and RMSD for various salt concentrations. For the salt concentrations of 1 M up to 10 M, the dynamic character of water molecules in the long time exhibits the diffusive motions in the both translation and rotation with the exponents of *α* = *β* = 1. It shows the same temporal characters of both dynamics as in bulk water. When the salt concentration further increases to 20 M, the situation is surprisingly different. We find that the TMSD exhibits the subdiffusive translational motion with the exponent of *α* = 0.69. In contrast, the RMSD consistently shows the diffusive motion with the exponent of *β* = 1, which is the same for all salt concentrations investigated. It means that the decoupling of the temporal characters in the dynamics would occur in the long-time behaviors of the translational and rotational motions in the superconcentrated limit, which has not been observed in bulk water. The translational and rotational dynamics of water molecules are closely related with how water molecules respond to their environments, suggesting that in the superconcentrated condition water molecules are differently coupled with their environments with respect with the translational and rotational dynamics. In general, the diffusive motion indicates a random process of dynamics represented by the Gaussian distribution, but the subdiffusive motion implies that dynamics is not random but non-trivially correlated. Thus, our results indicate that the translational dynamics becomes strongly correlated in the superconcentrated limit, whereas the rotational dynamics is still a random process.

The subdiffusive motion in the translation of water has been observed in a single-file diffusion inside a narrow quasi-one-dimensional carbon nanotube, representing the strongly correlated dynamics^31,32^. The relevant subdiffusive motions in the both translation and rotation of water, in contrast to our result, has been also proposed in nanotube rings^33^. Recently, an experimental study has found that the translational diffusion constant of water decreases upon the increasing pressure but the rotational diffusion constant insensitively responds to the pressure^34^. They ascribed it to the rigidity of the first neighboring shell of water and an invariance of the number of HBs. Even though the decoupled motion we found here is quite different from their result, the origin of different responses in the translation and rotation of water could be the same. The extremely ionic environments supply a severe drag to the translational motion of water molecules (similar to the rigidity) but still give the similar effect to the rotational motion by a contribution of HBs with anions (similar to the invariance of the HB number), as we will see later. One can also apply this interpretation to the single-file diffusion of water molecules. In the single-file diffusion, both dynamic origins are heavily affected by strong confinements, so that both the translational and rotational motions of water exhibit the same dynamic characters.

Using the TMSD and RMSD, we examine the translation diffusion constant *D*_*T*_ and the rotational diffusion constant *D*_*R*_ of water molecules. We calculate *D*_*T*_ from the TMSD via the Einstein relation^8,11,27,35^,

4 $$2d{D}_{T}t=\mathop{\mathrm{lim}}\limits_{t\to \infty }\langle {\rm{\Delta }}{\overrightarrow{r}}^{2}(t)\rangle ,$$

where *d* is the dimensionality of the system. Similarly, we calculate *D*_*R*_ from the RMSD^28,29,35,36^,

5 $$4{D}_{R}t=\mathop{{\rm{l}}{\rm{i}}{\rm{m}}}\limits_{t\to {\rm{\infty }}}\langle {\rm{\Delta }}{\overrightarrow{\varphi }}^{2}(t)\rangle .$$

If a molecule is linear, one angle would be sufficient to determine the rotational motion^28^. Since a water molecule is not the case, we also examine the other two normalized principal vectors denoted by $\overrightarrow{P}(t)$ and $\overrightarrow{Q}(t)$, as shown in Fig. 2(d). As the salt concentration increases, *D*_*T*_ decreases exponentially, which is caused by more viscous environments by increasing the number of ions. For the rotational motion, however, *D*_*R*_ for each principal vector shows different dependences on the salt concentration. Whereas *D*_*R*,*H*_ decreases upon increasing salt concentration, *D*_*R*,*P*_ and *D*_*R*,*Q*_ respond differently to the salt concentration. Both *D*_*R*,*P*_ and *D*_*R*,*Q*_ in the superconcentrated limit are larger than in bulk water, whereas *D*_*R*,*H*_ is smaller. Namely, the rotational motion with respect to one principal vector is suppressed but for the other vectors the rotational motions are enhanced. Unlike bulk water^29^, it suggests that the rotational motion of a water molecule is anisotropic in the superconcentrated limit. As we will see later, the oxygen atom of a water molecule strongly interacts with Li^+^ ions and thus the mobility of $\overrightarrow{H}(t)$ decreases upon increasing the number of salt. In contrast, the other two vectors, $\overrightarrow{P}(t)$ and $\overrightarrow{Q}(t)$, are affected mainly by the interaction of water molecules with anions as a HB donor. As a result, more ionic environments provide the smaller energy barrier of the rotations for water molecules with respect to $\overrightarrow{P}(t)$ and $\overrightarrow{Q}(t)$ but the larger energy barrier of the rotation with respect to $\overrightarrow{H}(t)$. It indicates that the increasing number of cations and anions induces anisotropy in the rotational dynamics of water molecules. Similar anisotropy in the rotational dynamics of a water molecules has been found in water in polymer networks^37^, suggesting the importance of cooperativity in ion hydration^14^. Note that Fig. 2(d) shows that *D*_*R*,*P*_ and *D*_*R*,*Q*_ initially decrease with increasing salt concentration. It explains that the initial increase of the salt concentration gives rise to the increasing interaction with anions, resulting in the initial decreases in *D*_*R*,*P*_ and *D*_*R*,*Q*_. For the further increase of the salt concentration, as we already discussed, the energy barrier of the rotations for water molecules with respect to $\overrightarrow{P}(t)$ and $\overrightarrow{Q}(t)$ becomes a dominant factor for the rotation.

### Hydrogen-bond dynamics and structure of water

**Figure 3:**
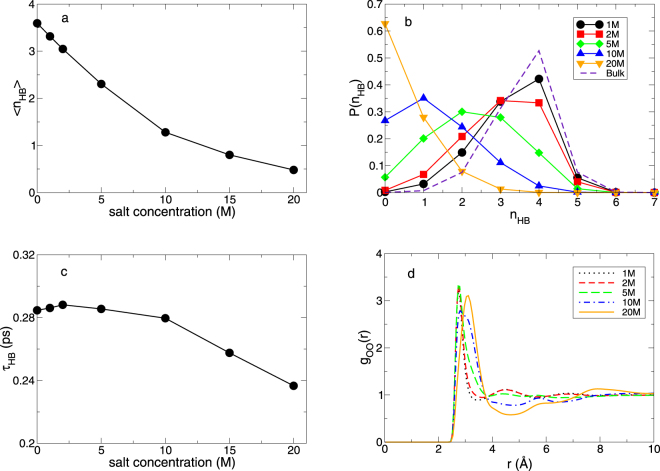
The hydrogen bond structure. (**a**) The average number 〈*n*_*HB*_〉 of hydrogen bonds per molecule between water molecules as a function of salt concentration. (**b**) The probability function *P*(*n*_*HB*_) having *n*_*HB*_ hydrogen bonds of a water molecule. (**c**) The average hydrogen bond lifetime *τ*_*HB*_ between water molecules as a function of salt concentration. (**d**) The oxygen-oxygen radial distribution function *g*_*OO*_(*r*) of water for various salt concentrations.

Due to the critical role of HBs in water, we need to investigate HBs of water molecules in various salt concentrations using the geometric definition of HB. The geometric definition describes that two tagged molecules are considered to be bonded if simultaneously their distance between two oxygens is less than 0.35 nm, and the angle between intra O-H and inter O $\cdots$ O is less than 30° ^4–6^. In Fig. 3(a), we calculate the average number 〈*n*_HB_〉 of the HBs per water molecule. 〈*n*_HB_〉 tends to decrease upon increasing salt concentration. At 20 M, 〈*n*_HB_〉 is 0.48 and it indicates that the majority of water molecules do not participate in the HB network of water in the superconcentrated limit. We can confirm the change in the HB structure from the probability function *P*(*n*_HB_) having *n*_HB_ hydrogen bonds per molecule, as shown in Fig. 3(b). We observe the noticeable deviation from bulk water at 5 M and *P*(*n*_HB_) at 20 M gives a totally different profile from bulk water. This gives a new feature in contrast to the previous report of negligible effects of (not superconcentrated) ions on the hydrogen bond network of water^38^. We infer that the difference in the HB network might come from the different range of salt concentrations and the different type of ions.

Next we calculate the average HB lifetime defined as

6 $${\tau }_{{\rm{HB}}}\equiv {\int }_{0}^{\infty }t{P}_{{\rm{HB}}}(t)dt\mathrm{.}$$

Here *P*_HB_(*t*) is the probability density function of the HB lifetime with the strict HB definition of the intact HB for the time interval^5,6^. The *τ*_HB_ is known to be related with the librational motion (the hidden rotation)^5,29^. After an initial increase by a small amount, *τ*_HB_ decreases upon further increasing salt concentration. It indicates that an intensity of HB between water molecules becomes much weaker in the superconcentrated limit. For the structure of water, the oxygen-oxygen radial distribution function (RDF) of water shows that the structural change in water occurs in the superconcentrated limit, as shown in Fig. 3(d). The first peak in the RDF occurs at *r* = 0.276 nm at bulk water and this is the same as in the salt concentrations from 1 M up to 5 M. As the salt concentration further increases, we observe the first peak in the RDF at *r* = 0.281 nm at 10 M and 0.311 nm at 20 M. Thus, the significant change in the structure of water is recognized from the salt concentration of 10 M.

To further examine the fast and slow kinetics of HBs of water molecules, we define continuous and discontinuous time distributions of HBs^4–6,39^. The former is the HB residence time distribution defined as

7 $${R}_{{\rm{HB}}}(t)\equiv \langle {\rm{\Theta }}({t}_{b}^{{\rm{HB}}}-t)\rangle ,$$

where Θ(*t*) is the step function and ${t}_{b}^{{\rm{HB}}}$ is the first-passage time for a HB to be broken. The latter is the HB correlation time distribution defined as

8 $${C}_{{\rm{HB}}}(t)\equiv \frac{\langle h(t)\cdot h\mathrm{(0)}\rangle }{\langle h\mathrm{(0)}\cdot h\mathrm{(0)}\rangle },$$

where *h*(*t*) is unity when the two tagged molecules are hydrogen-bonded at time *t* and *h*(*t*) is zero, otherwise. *C*_HB_(*t*) indicates the conditional probability that a HB remains intact at time *t*, given it was formed at time *t* = 0. *C*_HB_(*t*) does not consider any breaking of HB at intermittent times between time zero and *t*, whereas *R*_HB_(*t*) considers entirely intact HBs for the whole time interval. To quantify the characteristic time dependences of *R*_HB_(*t*) and *C*_HB_(*t*), we define two characteristic relaxation times, the characteristic HB residence time *τ*_*R*_ and the characteristic HB correlation time *τ*_*C*_, as the times required for *R*_HB_(*t*) and *C*_HB_(*t*) to decay by a factor of *e*, respectively.

**Figure 4:**
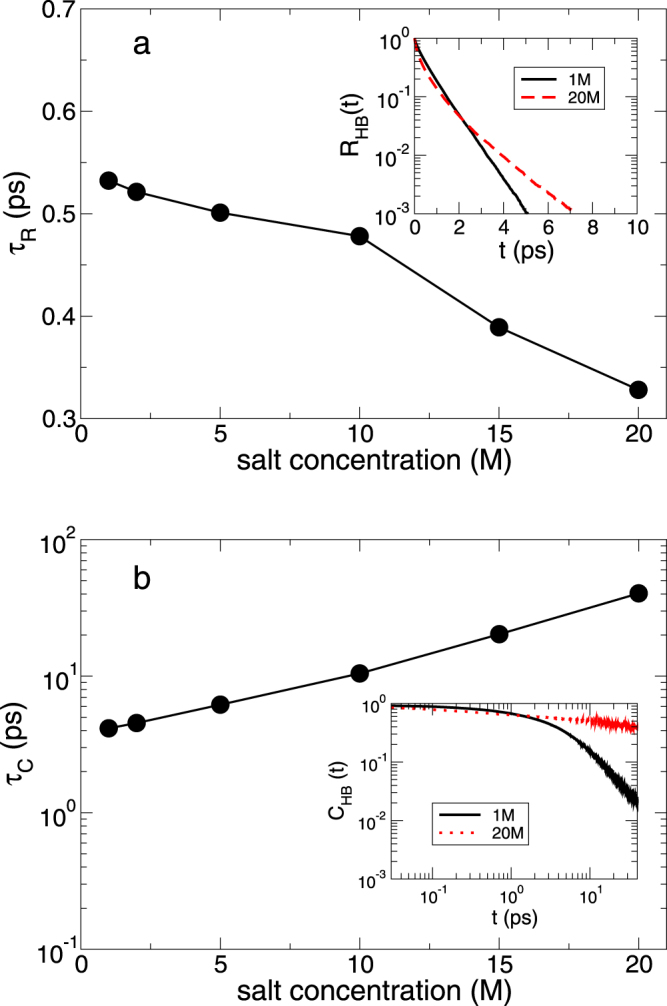
The fast and slow hydrogen-bond dynamics. (**a**) The characteristic HB residence time *τ*_*R*_ as a function of salt concentration. Inset: the HB residence time distribution *R*_*HB*_(*t*) in a semilog plot for the salt concentrations of 1 M and 20 M. (**b**) The characteristic HB correlation time *τ*_*C*_ as a function of salt concentration in a semi-log plot. Inset: the HB correlation time distribution *C*_*HB*_(*t*) in a log-log plot for the salt concentrations of 1 M and 20 M.

In Fig. 4, we find that *τ*_*R*_ and *τ*_*C*_ exhibit surprisingly different behaviors with respect to the salt concentration. For higher salt concentration, the *τ*_*R*_ becomes smaller but the *τ*_*C*_ is larger^26^. This result shows that there are two short and long timescales in association with different dependences on the salt concentration. Based on the connection of the fast dynamics of HB with the thermal vibration^5,6^, it means that higher salt concentration invokes faster thermal fluctuations in good agreement with the results of *D*_*R*,*P*_ and *D*_*R*,*Q*_. On the other hand, the slow dynamics of HB is closely related with the mass transport^6^ and the result of *τ*_*C*_ is also consistent with our results of the translational diffusion. Hence, importantly, this result implies that there are two different time scales in the HB dynamics of water^6,40^ in close relation to the translational and rotational dynamics.

### Solvation structure

**Figure 5:**
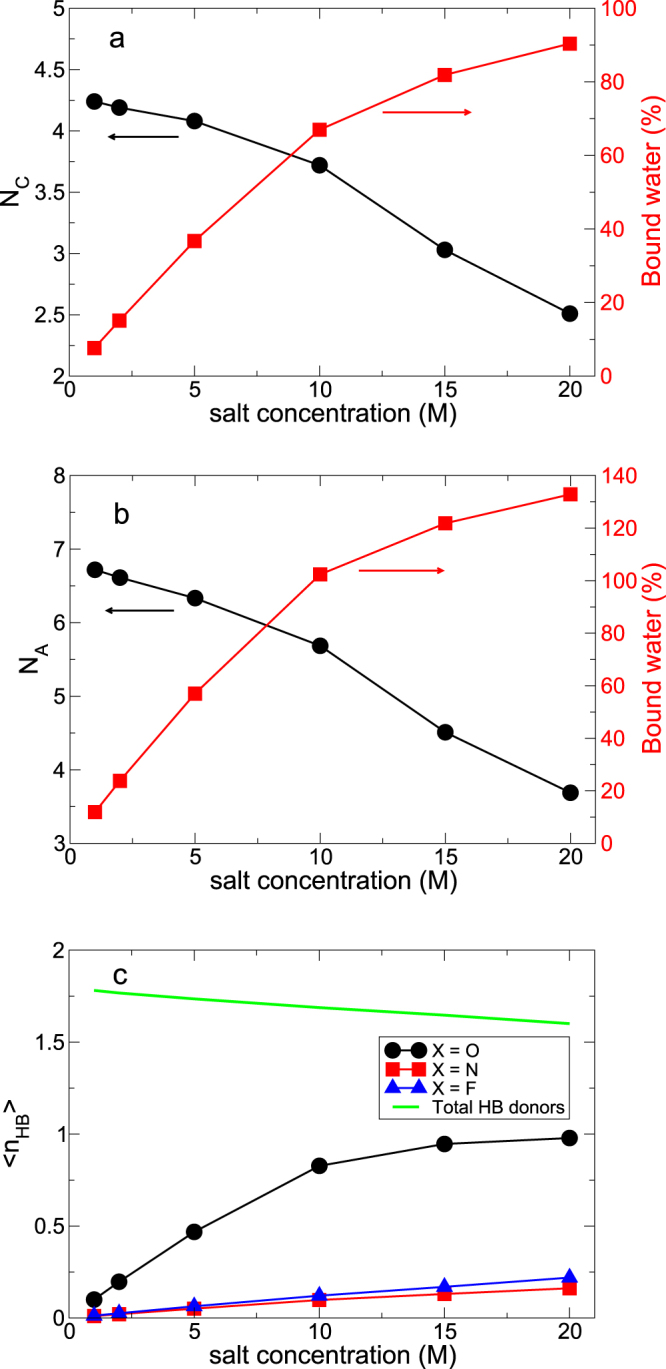
The cationic and anionic solvation structures. (**a**) The solvation number *N*_*C*_ of water molecules in the first solvation shell of a Li^+^ ion and the fraction of water molecules bound to Li^+^ ions. (**b**) The average number *N*_*A*_ of water molecules hydrogen-bonded with a TFSI^−^ anion and the fraction of water molecules bound to TFSI^−^ ions. (**c**) The average HB number per water molecule with anions, O-H... X^−^, where X is an tagged atom of an anion. The green solid line presents the average number of total HB donors (to water and anions) of a water molecule.

The dynamics of water is strongly affected by the existence of ions, so that it is necessary to explore the interaction between salt and water. First, we calculate the Li solvation number *N*_*C*_ of water (Fig. 5). We define *N*_*C*_ as the number at the first plateau in the cumulative coordination number $n(r)\equiv 4\pi \rho {\int }_{0}^{r}{r}^{^{\prime} 2}{g}_{{\rm{LW}}}(r^{\prime} )dr^{\prime}$, where *g*_LW_(*r*) is the RDF between Li^+^ ions and water molecules^21^. At 1 M, the water molecules of *N*_*C*_ = 4.24 are found in the solvation sheath of a Li^+^ ion. As the salt concentration increases to 20 M, the number of water molecules in the solvation shell decreases to *N*_*C*_ = 2.51. By classifying water molecules into those in the solvation sheath (*bound*) and the other outside it (*free*), we find that the fraction of the bound water molecules increases from 7.6% at 1 M up to more than 90% at 20 M. Thus, most water molecules interact with cations in the superconcentrated limit, as shown in Fig. 1.

For a TFSI^−^ ion, we calculate the average number *N*_*A*_ of water molecules hydrogen-bonded with an anion due to the ionic size. Similarly, we refer to a water molecule as bound if the molecule is hydrogen-bonded with an anion and the molecule is referred to as free, otherwise. The fraction of the water molecules bound to an anion is around 12% at 1 M and exceeds 100% at 20 M, indicating that a significant portion of water molecules are hydrogen-bonded with more than two anions at 20 M. It means that the interaction between salt and water becomes more dominant than the interaction between water molecules in the superconcentrated limit, as shown in Fig. 1. Due to the increasing interaction of water molecules with anions, the number of HBs with anions naturally increases with the increasing salt concentration.

Water molecules simultaneously act as a HB acceptor as well as a HB donor to each other. As for anions, water molecules mostly play the role of a HB donor to them^14^. Hence the total HB donor number of water molecules would provide an important factor to explain any environmental changes for the rotational dynamics of water molecules. Figure 5 shows that only a subtle decrease in the total HB donor number of water molecules is observed as the salt concentration increases from 1 M up to 20 M. The similar environments remain for the rotational motion of water molecules by keeping the (almost) same number of HB donors. It is consistent with our interpretation of the decoupled temporal character between the translational and rotational motions.

### Dynamics and solvation structure of a Li^+^ ion

**Figure 6:**
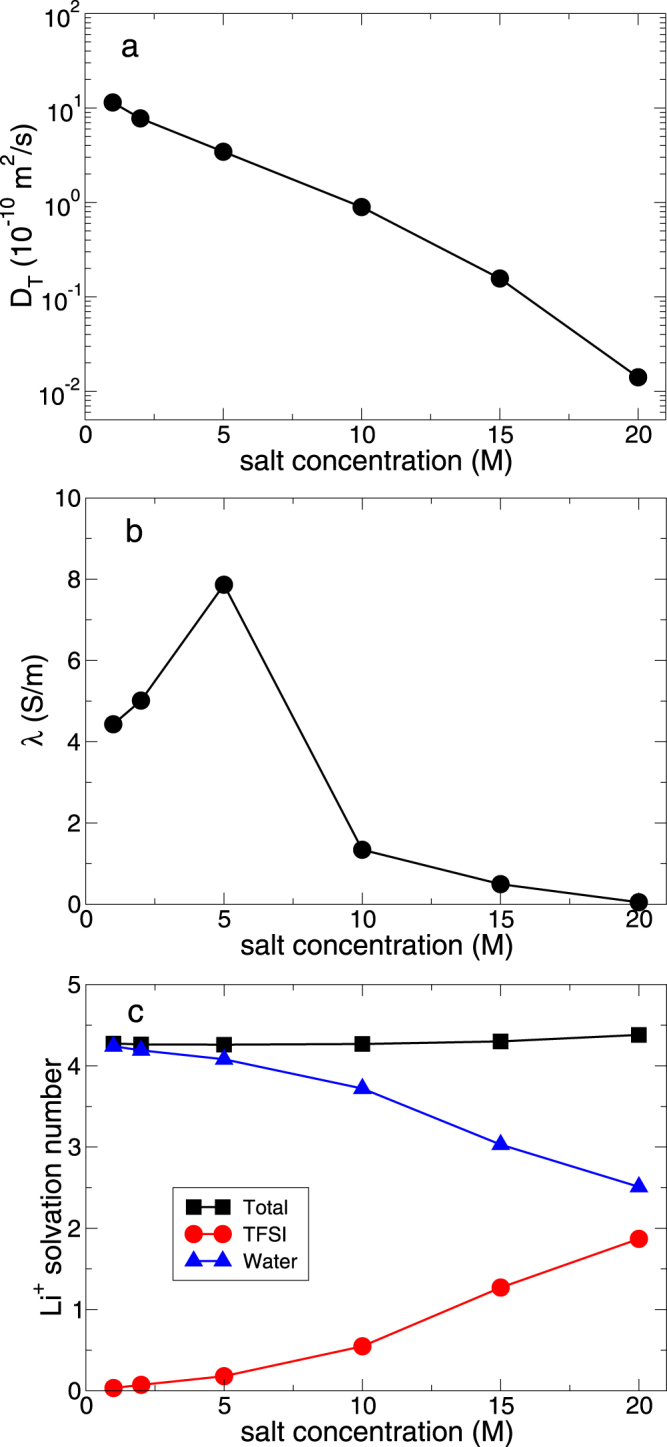
Dynamics and solvation structure of Li^+^ ions. (**a**) Translational diffusion constant *D*_*T*_ of a Li^+^ ion as a function of salt concentration. Note that *D*_*T*_ at 20 M is not real diffusion constant due to the subdiffusive nature, but it is just an estimate the same as water in Fig. 2. (**b**) Ionic conductivity λ as a function of salt concentration. (**c**) Total solvation number in the first shell of a Li^+^ ion as a function of salt concentration. We also present the degree of salt association as a function of salt concentration.

At last, we consider the dynamics and solvation structure of a Li^+^ ion. For the dynamics of a Li^+^ ion, we calculate the translational diffusion constant of a Li^+^ ion and the ionic conductivity. As shown in Fig. 6(a), the translational diffusion constant of a Li^+^ ion exhibits the similar behavior to one of a water molecule. As the salt concentration increases, the diffusion constant exponentially decays. Next, we calculate the ionic conductivity λ defined as^21^

9 $$6tV{k}_{{\rm{B}}}T{\rm{\lambda }}=\mathop{\mathrm{lim}}\limits_{t\to \infty }\sum _{i}^{N}\sum _{j}^{N}{z}_{i}{z}_{j}{e}^{2}\,\langle [{{\bf{r}}}_{i}(t+{t}_{0})-{{\bf{r}}}_{i}({t}_{0})]\cdot [{{\bf{r}}}_{j}(t+{t}_{0})-{{\bf{r}}}_{j}({t}_{0})]\rangle ,$$

where *z* is the charge of an ion in the unit of the elementary charge *e*. The summations are over all ions of the system. In Fig. 6(b), the ionic conductivity λ shows the maximum around the salt concentration of 5 M, which is in agreement with the recent experiment^25^.

We further consider the solvation structure of a Li^+^ ion and the degree of the salt aggregation as a function of salt concentration. In Fig. 6(c), we present the solvation number of a Li^+^ ion. The total solvation number is around 4.273 at 1 M, 4.260 at 5 M, and 4.380 at 20 M. Thus, the total solvation number does not show the strong dependence on the salt concentration. As the salt concentration increases, the water molecules in the solvation shell are replaced by the same number of anions. The number of anions in the solvation shell gradually increases: it is below 1.0 up to 10 M and increases to 2.0 at 20 M. It indicates that a salt aggregation occurs in the superconcentrated conditions.

## Conclusion

The effects of ions on the structure and dynamics of water are of importance in a wide range of systems. Most questions have been raised to explore a degree of deviations in the structural and dynamic properties of water from bulk properties in the presence of ions. A lot of efforts have been thus made to elucidate what properties of ions would be key to explain the effects of ions on water and what would be the mechanism for ions to contribute to variations in the properties of water. Some studies have focused on the effects of different ions on the structure and dynamics of water. They have also looked into the effects of the ionic concentrations up to the moderate level. However, the extremely high concentrated condition of salt have not been considered mainly due to the lack of its applications.

Recently, pioneering experiments open a possibility of new applications of superconcentrated aqueous electrolytes to lithium ion batteries. In spite of the recent progress, little is known about the properties of water in the extremely high salt concentration. Hence we have investigated the dynamics of water in aqueous electrolytes for various salt concentrations. We have found that the dynamic and structural features of water in the superconcentrated limit exhibit new and unusual behaviors compared to bulk properties of water. We have observed that there is the decoupled temporal character between the translational and rotational dynamics, indicating that only one of the two origins related with the dynamics is strongly affected by the superconcentrated environments. Furthermore, the superconcentrated condition induces the anisotropic rotation of water molecules, which is closely related with the fact that the oxygen atom of a water molecule interacts with cations and the hydrogen atom interacts with anions. The anisotropy in rotational dynamics of water molecules has been found in polymer networks^37^. It indicates the importance of the cooperativity in ion hydration and suggests the possibility of finding the similar anisotropy of the rotational dynamics at various environments. Those new dynamic features continue to the HB characteristics of water such as the emergence of two different timescales in the dependence on the salt concentration of the HB dynamics.

In this work, we have found that water in superconcentrated environments shows many interesting features different from bulk water. We believe that the new dynamic features of water molecules we found here will give the better understanding of superconcentrated aqueous electrolytes, continuing to give a direction for a new design of future batteries. Also, we believe that our results on the dynamics of water in superconcentrated environments will broaden the understanding of the properties of water in ionic environments.

## Methods

We perform molecular dynamics (MD) simulations of aqueous electrolytes of lithium ion batteries consisting of a solution of a lithium bis(trifluoromethane sulfonyl)imide (LiTFSI) salt in water modeled with the extended simple point charge (SPC/E) model^41^. We investigate the systems with the five different salt concentrations: 1, 2, 5, 10, 15 and 20 M. The number of water molecules is *N*_*W*_ = 5832 and the number of the LiTFSI salt is *N*_*S*_ = 105 up to 2100, depending on the salt concentration. For bulk water, we use *N*_*W*_ = 8000. We carry out all simulations using the MD simulation package, LAMMPS^42^. We implement the OPLS/AA force field to describe the molecular interaction of the LiTFSI salt^43^ and the force field parameters of the SPC/E water model can be found in ref.^41^. We use the combination rule of the Lorentz–Berthelot for the intermolecular interactions of Li^+^–TFSI, TFSI–H_2_O and Li^+^–H_2_O. We compute the long-range interactions using particle-particle particle-mesh (PPPM) algorithm. The simulations are performed initially in the *NPT* ensemble and then in the *NVT* ensemble, where *N*, *V*, *P* and *T* are the number of molecules (*N*_*W*_ + *N*_*S*_), the volume, the pressure and the temperature, respectively. We keep the temperature and pressure constant via the Nóse-Hoover thermostat and barostat during the simulations. We apply periodic boundary conditions in all three directions of the simulation box. We use 1 fs as a timestep of the simulation. For each salt concentration, we run the MD simulations of 50 ns for the equilibration and 30 ns for the data collection. Initially, we prepare the random configuration, and then increase the temperature up to 400 K to mix the system properly. After then, we decrease the temperature the target temperature, 300 K, and equilibrate the system. We repeat the MD simulations 3-4 times with different initial conditions.

### Data availability

The datasets generated during and/or analysed during the current study are available from the corresponding author on reasonable request.
